# High molecular weight hyaluronan decreases UVB-induced apoptosis and inflammation in human epithelial corneal cells

**Published:** 2009-03-23

**Authors:** Thierry Pauloin, Mélody Dutot, Francine Joly, Jean-Michel Warnet, Patrice Rat

**Affiliations:** 1 Laboratoire de Toxicologie, Faculté des Sciences Pharmaceutiques et Biologiques, Université Paris Descartes, Paris, France; 2 SEPhRA (Société d'Etudes en Pharmacologie: Recherche, Applications), Puteaux, France; 3 INSERM, UMR S 872, Institut Biomédicale des Cordeliers, Université Paris Descartes, Paris, France

## Abstract

**Purpose:**

The aim of this study was to investigate high molecular weight hyaluronan (HMW-HA) protection on human corneal epithelial (HCE) cells against ultraviolet B (UVB) radiation-induced toxic effects.

**Methods:**

The HCE cell line was incubated with HMW-HA or phosphate-buffered salt solution (PBS), rinsed, and exposed to UVB radiation. Cell viability, reactive oxygen species (ROS) and glutathione (GSH) levels, 8-hydroxy-2'-deoxyguanosine (8-oxo-dG) release, p53 phosphorylation, caspase-3, -8, -9 activation, and interleukin (IL)-6 and -8 production were assessed to evaluate and to compare UVB-induced toxicity between cells treated with HMW-HA and cells treated with PBS.

**Results:**

Data indicate that HMW-HA had significant protective effects against UVB radiation. HMW-HA increased HCE cell viability, decreased IL-6 and -8 production, and decreased caspase-3 and -8 activation. However, HMW-HA had no significant effect on ROS and GSH levels, 8-oxo-dG release, and p53 phosphorylation.

**Conclusions:**

To our knowledge, we report for the first time the ability of HMW-HA to protect cells against UV irradiation. According to our results, HMW-HA provides anti-inflammatory and anti-apoptotic signals to cells exposed to UVB.

## Introduction

Ultraviolet (UV) light is the most common cause of radiation injury to the eye. Under normal conditions, the cornea absorbs the majority of UVB (280–320 nm) rays and protects the inner eye against UVB-induced damaging effects. Exceeded threshold levels of UVB light absorption induce inflammation of the cornea (photokeratitis), which typically appears 6–12 h after exposure [1]. Symptoms include tearing, pain, redness, swollen eyelids, headache, a gritty feeling in the eyes, halos around lights, hazy vision, and temporary loss of vision. These symptoms may not appear until 6–12 h after the UVB exposure.

Cells exposed to UVB radiation show excessive levels of reactive oxygen species (ROS), DNA damage, activation of death receptors, and secretion of inflammatory cytokines, which all contribute to UVB-induced apoptosis [2]. ROS-mediated oxidative damage causes DNA modification, lipid peroxidation, and secretion of inflammation cytokines [3]. In DNA, 2'-deoxyguanosine is easily oxidized by ROS to 8-hydroxy-2'-deoxyguanosine (8-oxo-dG) [4]. 8-oxo-dG is a substrate for several DNA based excision repair systems and is released from cells after DNA repair. This modified nucleobase is used extensively as a biomarker of oxidative damage in DNA. As a transcription factor, the p53 protein is phosphorylated and activated in response to UVB-induced DNA damage. p53 activates genes that can either arrest cell cycle progression, which can provide sufficient time to repair damaged DNA, or initiate programmed cell death (apoptosis) [5]. The UVB-induced apoptotic process involves both extrinsic and intrinsic pathways [6]. The extrinsic pathway is triggered by cell death membrane receptor activation followed by the activation of caspase-8. The intrinsic pathway is triggered by mitochondrial death signals, which lead to the activation of caspase-9. Activation of these initiator caspases (caspase-8 and -9) via either pathway activates caspase-3 that cleaves a variety of cellular death subtrates leading to apoptosis [7].

Hyaluronan (HA) is a linear polymer non-sulfated polysaccharide chain composed of alternating β-1,4-glucuronic acid and β-1,3-N-acetylglucosamine [8]. HA is synthesized as large disaccharide chains (>10^6^ Da), which is progressively degraded in the extracellular matrix [9]. HA interacts specifically with several cell surface receptors including CD44, which is considered the main HA cell-surface receptor [10]. In the eye, HA is mainly distributed in the vitreous body of the eye. However, it is not present in lachrymal film or in tears. HA is used in eye drops for dry eye syndrome. Dry eye syndrome is generally associated with a disorder of the lachrymal film, which is accompanied by various symptoms of ocular discomfort. In this case, HA is used for its ability to create a hydrophilic gel and therefore to stabilize the lachrymal film. In a previous study, we showed that HA and specifically high molecular weight hyaluronan (HMW-HA) protects human corneal epithelial (HCE) cells against cytotoxicity of benzalkonium chloride, which is one of the most often used preservative in aqueous pharmaceutical products [11]. Moreover, in a recent study, we showed that HMW-HA significantly decreased rabbit ocular surface damages induced by sodium lauryl sulfate instillation [12]. However, it was not described how HMW-HA acts and what the implicated mechanisms were.

Our study was performed to demonstrate whether HMW-HA is an active agent promoting cell protection and what the implicated mechanisms are. HA sugar residues absorbed UV radiations at 230–240 and 260–270 nm [13]. To investigate whether HMW-HA actively promotes cell protection, HCE cells were incubated with hyaluronan and exposed to UVB radiation (~312 nm) that cannot be absorbed by HMW-HA. Cell viability, oxidative stress, apoptosis, DNA damage, and inflammation were then evaluated.

## Methods

### Cell culture

A human corneal epithelial cell line [14] (HCE, RCB-1384; Riken Cell Bank, Tsukuba, Japan) was cultured in Dulbecco’s minimum essential medium mixed with Ham’s F12 medium (in a 50:50 ratio; Eurobio, Les Ulis, France) supplemented with 10% fetal bovine serum (Eurobio), 1% glutamine (Eurobio), and 1% penicillin and streptomycin (Eurobio). Cultures were maintained at 37 °C in 5% CO_2_ in a humidified incubator.

### Cell incubation

All of the wells were seeded with the same number of cells (9×10^4^ cell/ml solution). After 24 h, cells reached approximately 70%–80% confluence. Cells were incubated 30 min with 0.2% (w/v) HMW-HA (1.5 10^6^ Da; Soliance, Pomacle, France) or with phosphate-buffered salt solution (PBS; Eurobio). Cells were then washed with PBS and irradiated with 25, 75, 150, and 200 mJ/cm^2^ of UVB (~312 nm; Vilber Lourmat, Marne La Vallée, France). All further tests except ROS and GSH quantification were performed after a 24 h recuperation time in the culture medium. Experiments were performed at least in triplicate.

### Cell viability

The neutral red (Fluka, Buchs, Switzerland) uptake assay is a cell viability assay based on the ability of viable cells to incorporate neutral red, which is a weak cationic dye that readily penetrates cell membranes by non-ionic diffusion and accumulates in lysosomes where it binds with anionic sites in the lysosomal matrix. Lysosomal membrane integrity is closely correlated with cell viability and is evaluated with neutral red fluorescence (excitation, 535 nm; emission, 600 nm) [15,16]. Neutral red solution (50 μg/ml) diluted in the above culture medium was added to living cells. After a 3 h incubation at 37 °C, cells were washed with PBS and incubated with a lysis solution (1% acetic acid, 50% ethanol, and 49% H_2_O). The plate was agitated on a microplate shaker for 20 min, and then fluorescence was measured using microplate fluorometry (Safire; Tecan, Lyon, France).

### Reactive oxygen species production

2',7'-Dichlorodihydrofluorescein diacetate (H_2_DCF-DA; Invitrogen, Carlsbad, CA) is a standard probe to detect reactive oxygen species in cells. It is colorless and nonfluorescent until the cleavage of the diacetate group. It is a non-polar compound, which is hydrolyzed by intracellular esterases to become a nonfluorescent polar derivative (H_2_DCF). This nonfluorescent polar derivative is oxidized rapidly to give the highly green fluorescent, 2’,7’-dichlorofluorescein, in the presence of intracellular reactive oxygen species [17]. Fluorescence (excitation, 490 nm; emission 535 nm) was measured after a time incubation of 20 min.

### Quantification of the intracellular glutathione

Monochlorobimane fluorogen probe (Invitrogen) was used to evaluate intracellular glutathione levels. It is nonfluorescent until it reacts with a thiol to form a blue fluorescent product, which which allows the quantification of intracellular GSH. Fluorescence (excitation, 380 nm; emission 460 nm) was measured after an incubation time of 30 min.

### Caspase-3, -8, and -9 activation

Activation of caspase-3, -8, or -9 was detected using the Fluorometric Assay Kit according to the manufacturer’s instructions (Biovision, Mountain View, CA). In brief, control or treated cells were lysed in 50 µl of cold lysis buffer and kept on ice for 10 min. Cell lysate was added to 50 µl of reaction buffer and 5 µl of fluorogenic report substrates specific for caspase-3 (DEVD [aspartic acid-glutamic acid-valine-aspartic acid]-7-amino-4-trifluoromethylcoumarin [AFC]), caspase-8 (IETD [isoleucine-glutamic acid-threonine-aspartic acid]-AFC), or caspase-9 (LEHD [leucine-glutamic acid-histidine-aspartic acid]-AFC). After incubation at 37 °C for 2 h, AFC fluorescence (excitation, 400 nm; emission, 505 nm) was measured with a fluorescence microplate reader (Safire; Tecan). Comparison of the absorbance from cells with and without treatment allows the determination of relative caspase activation.

### Quantification of 8-hydroxy-2’-deoxyguanosine

 The concentration of released 8-oxo-dG in the cell culture supernatant of HCE cells was determined by enzyme-linked immunosorbent assay (ELISA). Twenty-four hours after UVB irradiation, HCE cell culture supernatants were harvested, centrifuged, and stored at −80 C until use. The concentration of 8-oxo-dG excreted by HCE cells was measured according to the manufacturer’s instruction (Trevigen, Gaithersburg, MD).

### Quantification of Phospho-p53 Ser 46

The analysis of p53 serine 46 was performed 24 h after UVB irradiation using a Phospho-p53 Ser 46 ELISA Kit (Cyclex Co., Nagano, Japan) according to the manufacturer’s protocol. A biotinylated detection antibody specific for human p53 phosphorylated at serine 46 in cell lysates was used to detect phosphorylated protein with a standard streptavidin-HRP method. The results were read at 450 nm.

### Determination of IL-6 and IL-8 concentrations

The concentration of released cytokines in the cell culture supernatant of HCE cells was determined by ELISA. Twenty-four hours after UVB irradiation, HCE cell culture supernatants were harvested, centrifuged, and stored at −80 C until used for cytokine measurements. The concentration of cytokines released by HCE cells was measured according to the manufacturer’s instructions for IL-6 (eBioscience, San Diego, CA) and IL-8 (Raybiotech, Norcross, GA) and was adjusted by the number of remaining cells.

### Statistical analysis

Analysis was performed using Sigma Stat 2.0 (Chicago, Illinois). All data are expressed as the mean±standard error of mean. ANOVA for comparison of the different groups was used with significance set at p<0.05. A significant ANOVA was followed by a Fisher test for multiple comparisons between groups, significance was set at p<0.05.

## Results

### HMW-HA significantly decreased UVB-induced cell death

**Figure 1:**
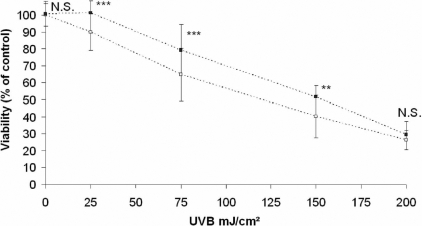
Effect of hyaluronan on HCE cell viability after UVB exposure. HCE cells were incubated with PBS (clear square) or HMW-HA (black square) and irradiated with various doses (0, 25, 75, 150, and 200 mJ/cm^2^) of UVB. Cell viability was determined by neutral red uptake assay 24 h after irradiation. Data are mean±SD of nine independent experiments. Differences were significant at p<0.05 (one asterisk), p<0.01 (two asterisks), and p<0.001 (three asterisks) compared to the PBS group of each UVB dose.

HCE cell viability was evaluated 24 h after irradiation. As showed in Figure 1, at dose 25, 75, 150, and 200 mJ/cm² of UVB, cell viability significantly decreases in a dose-dependant manner (90%, 64%, 40%, and 26%, respectively). At dose 25, 75, and 150 mJ/cm^2^ of UVB, cells treated with HMW-HA were significantly less susceptible to UVB radiation (100%, 79%, and 51%, respectively) compared with control cells. These doses were selected for further experiments.

### HMW-HA had no significant effect on UVB-induced oxidative stress

**Figure 2:**
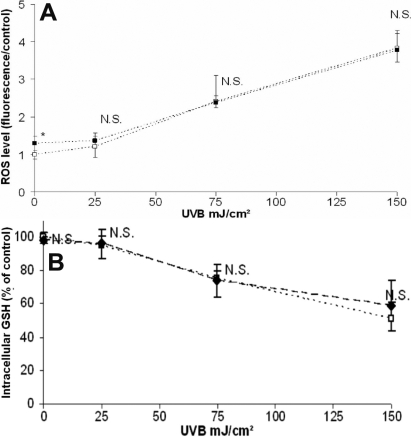
Analysis of intracellular ROS and GSH levels following UVB irradiation. HCE cells were incubated with PBS (clear square) or HMW-HA (black square) and irradiated with various doses (0, 25, 75, and 150 mJ/cm^2^) of UVB. The intracellular ROS level (**A**) was measured with a H_2_DCF-DA fluorogen probe, and the intracellular GSH level (**B**) was measured with a monochlorobimane fluorogen probe after UVB irradiation. Results are expressed as mean±SD of three independent experiments. Differences were significant at p<0.05 (one asterisk), p<0.01 (two asterisks), and p<0.001 (three asterisks) compared to the PBS group of each UVB dose.

UVB radiation (25, 75, and 150 mJ/cm^2^ of UVB) induced increases in ROS levels (+21%, +240%, and +384%, respectively) and a correlated decrease in GSH levels (−7%, −23%, and −49%, respectively) in a dose-dependant manner, showing that UVB induced stress oxidants in HCE cells (Figure 2). At the selected UVB doses, we did not observe any difference in UVB-induced oxidative stress between cells treated with HMW-HA and control cells. HMW-HA induced a slight ROS production without significant difference in GSH level in non-irradiated cells compared to control cells.

### HMW-HA significantly decreased UVB-induced caspases-3 and -8 activation but not caspase-9 activation

**Figure 3:**
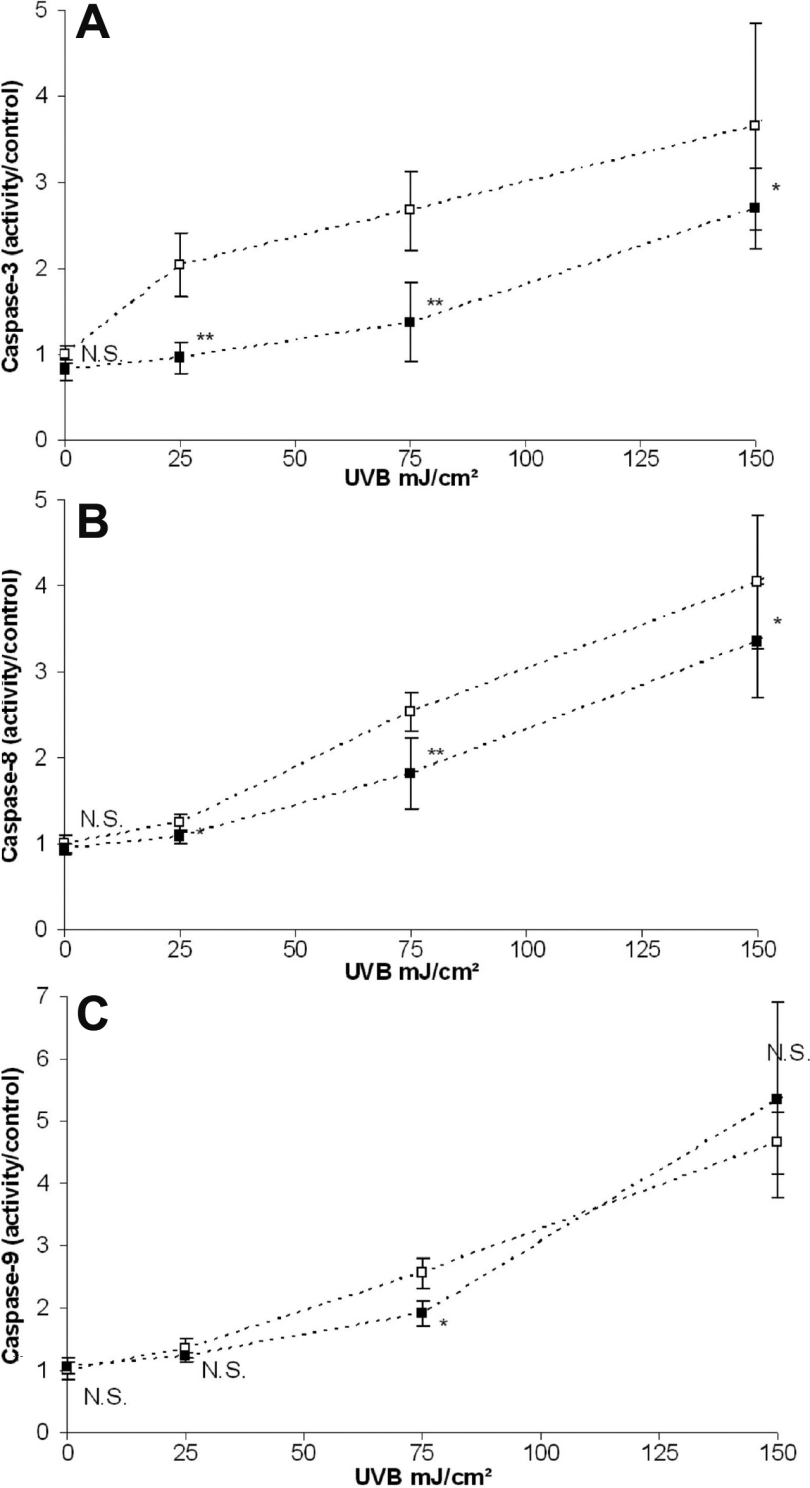
Analysis of caspase-3, -8, and -9 activation. HCE cells were incubated with PBS (clear square) or HMW-HA (black square) and irradiated with various doses (0, 25, 75, and 150 mJ/cm^2^) of UVB. Caspase-3 (**A**), -8 (**B**), and -9 (**C**) activations were determined 24 h after irradiation. Data are mean±SD of three independent experiments. Differences were significant at p<0.05 (one asterisk), p<0.01 (two asterisks), and p<0.001 (three asterisks) compared to the PBS group of each UVB dose.

We investigated caspases-3, -8, and -9 activation 24 h after UVB irradiation. At doses of 25, 75, and 150 mJ/cm^2^ of UVB, caspases-3, -8 and -9 were activated in a dose-dependant manner (Figure 3). The UVB-induced apoptotic process of HCE cells involved both caspase-8 and caspase-9 pathways. At doses of 25, 75, and 150 mJ/cm^2^ of UVB, cells treated with HMW-HA showed a clear decrease in the activation of caspase-3 (−52%, −48%, and −26%, respectively) and caspase-8 (−14%, −57%, and −39%, respectively) compared with control irradiated cells. HMW-HA completely prevented caspase-3 and caspase-8 activation at the dose of 25 mJ/cm^2^ of UVB. Caspase-9 activation was quite similar in both PBS and HMW-HA treatments, except at the dose 75 mJ/cm^2^ of UVB, which indicated a significant caspase-9 decrease (−47%) with HMW-HA treatment.

### HMW-HA did not affect UVB-induced DNA damage and p53 activation

**Figure 4:**
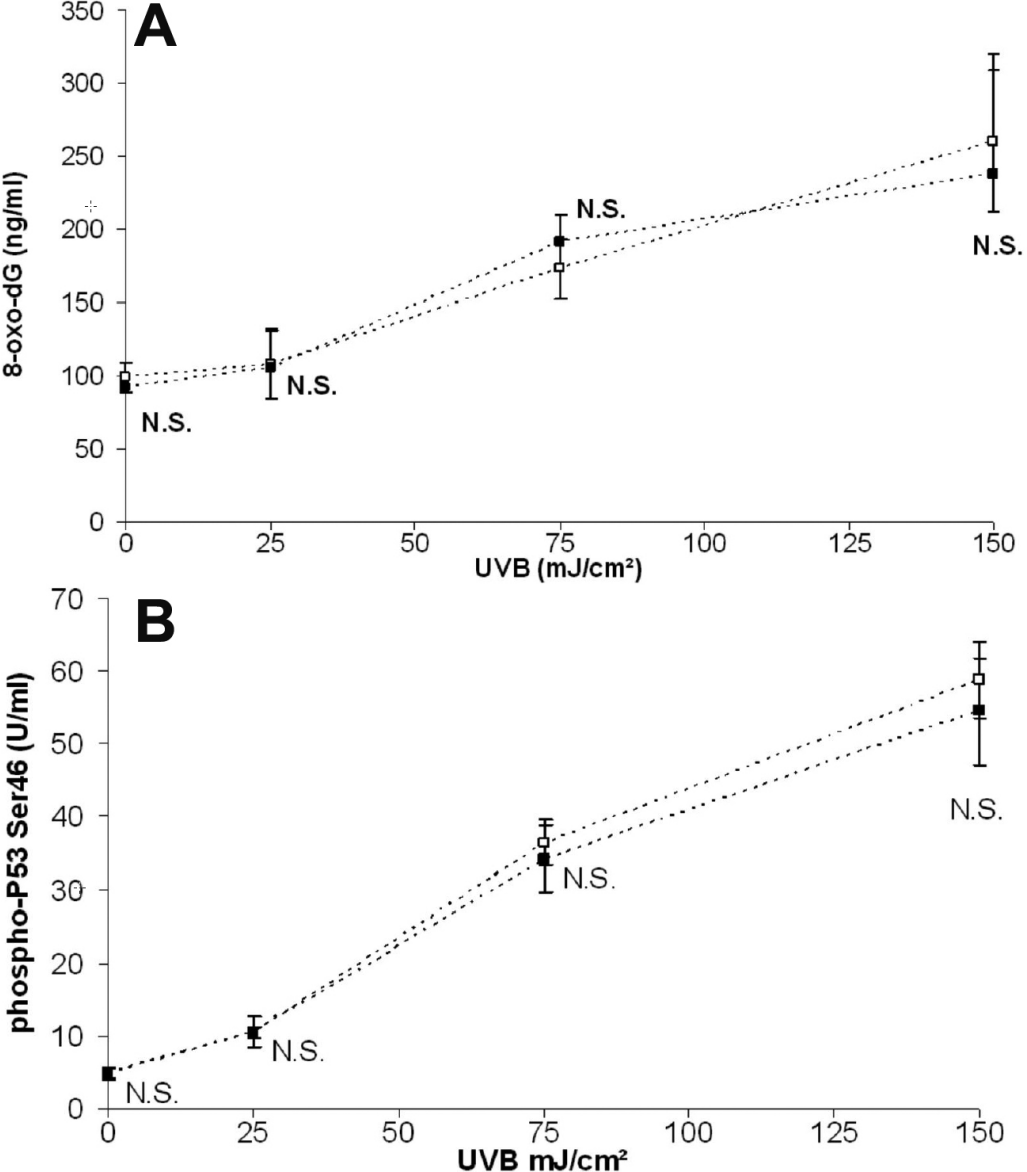
Analysis of 8-oxo-dG levels and p53 phosphorylation. HCE cells were incubated with PBS (clear square) or HMW-HA (black square) and irradiated with various doses (0, 25, 75, and 150 mJ/cm^2^) of UVB. The concentration of 8-oxo-dG excreted in the cell culture supernatant and quantification of serine 46-phosphorylated p53 were determined by ELISA 24 h after irradiation. **A**: The concentration of 8-oxo-dG excreted in the cell culture supernatant and **B**: quantification of serine 46-phosphorylated p53 were determined by ELISA 24 h after irradiation.Data are mean±SD of three independent experiments. Differences were significant at p<0.05 (one asterisk), p<0.01 (two asterisks), and p<0.001 (three asterisks) compared to the PBS group of each UVB dose.

We evaluated UVB-induced DNA damage with 8-oxod-dG quantification in HCE cell supernatant and p53 activation 24 h after UVB irradiation. As shown in Figure 4, UVB radiation (25, 75, and 150 mJ/cm^2^ of UVB) induced increases in 8-oxo-dG release (+12%, +75%, and +163%, respectively) and in p53 phosphorylation (+102%, +630%, and +710%, respectively) in a dose-dependant manner. At the selected UVB doses, we did not observe any effect of HMW-HA in 8-oxo-dG production or p53 phosphorylation compared with HCE control irradiated cells.

### HMW-HA significantly decreased UVB-induced IL-6 and IL-8 production

**Figure 5:**
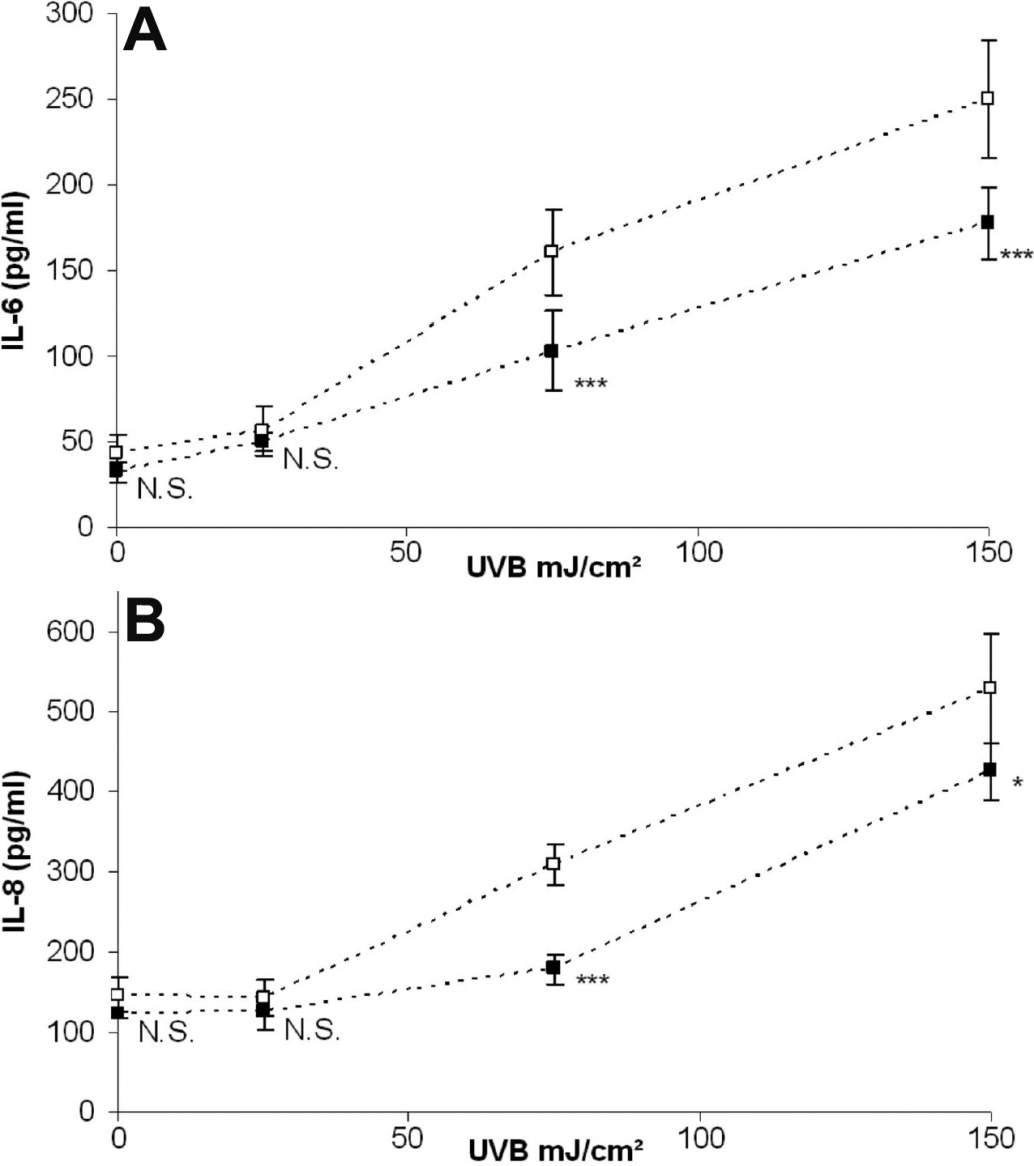
Analysis of secreted IL-6 and IL-8 cytokine concentrations. HCE cells were incubated with PBS (clear square) or HMW-HA (black square) and irradiated with various doses (0, 25, 75, and 150 mJ/cm^2^) of UVB. The concentration of secreted IL-6 (**A**) and IL-8 (**B**) cytokines in the cell culture supernatant of HCE cells was determined by ELISA 24 h after irradiation. Data are mean±SD of three independent experiments. Differences were significant at p<0.05 (one asterisk), p<0.01 (two asterisks), and p<0.001 (three asterisks) compared to the PBS group of each UVB dose.

The determination of inflammation cytokine production in HCE cell supernatant showed that at 75 and150 mJ/cm^2^ of UVB, IL-6 production (Figure 5A) increased in a dose-dependant manner and is significantly decreased with HMW-HA (−36% and −29%, respectively). At 75 and 150 mJ/cm^2^ of UVB, IL-8 production (Figure 5B) increased in a dose-dependant manner and is significantly decreased with HMW-HA (−42% and −20%, respectively).

## Discussion

UVB radiation, which is known to damage corneal epithelial cells, contributes to ocular pathologies that include photokeratitis. The objective of this study was to determine whether high molecular weight hyaluronan (HMW-HA) protects human corneal epithelial cells (HCE) against UVB radiation. The neutral red uptake assay was performed to evaluate differences in cell viability between HCE cells treated with HMW-HA and control cells treated with phosphate-buffered salt solution before irradiation with classic doses (25, 75, 150, and 200 mJ/cm^2^) of UVB. We showed that these doses of UVB radiation on HCE cells decreased cell viability. From 25 to 150 mJ/cm^2^ of UVB, we observed that HCE cells treated with HMW-HA were less susceptible to UVB radiation, demonstrating a protective effect of HMW-HA. These results were particularly interesting because HA is a biopolymer composed of sugar residues, which absorbed UV radiations at 230–240 and 260–270 nm [13]. The UVB irradiation (~312 nm) that we induced can not be absorbed by HMW-HA.

Oxidative stress, DNA damage, and death receptor activation independently participate in the formation of UVB-induced apoptotic cells [18,19]. To investigate the HMW-HA protective mechanism, we analyzed separately UVB-induced oxidative stress, DNA damage, and death receptor activation, notably with caspase-8 activation. Oxidative stress is caused by an imbalance between the production of ROS and a biological system's ability to readily detoxify the reactive intermediates. Glutathione (GSH), which is one of the most effective intracellular antioxidants, exists in reduced and oxidized (GSSG) states. An increased GSSG-to-GSH ratio is considered indicative of oxidative stress. To examine the oxidative stress induced by UVB, analysis of intracellular ROS formation and quantification of intracellular GSH levels was performed. HCE exposed to UVB exhibited an increase in ROS production and a decrease in GSH levels. The imbalanced state between the production of ROS and antioxidant defenses characterized UVB-induced oxidative stress. We then assessed the effect of HMW-HA against UVB-induced oxidative stress. Our findings did not show any antioxidative effect of HMW-HA.

Excessive levels of ROS induced apoptosis in a variety of cell types by inducing DNA damage [20]. In this study, we assessed DNA damage with 8-oxo-dG, a modified base, which is the most commonly studied and detected by-product of DNA damage that is excreted upon DNA repair [21]. DNA damage can change phosphorylation levels of the p53 protein, resulting in cell cycle arrest and apoptosis [22,23]. Present data indicate that UVB radiation caused DNA damage and p53 activation in HCE cells. No significant difference was observed with cells treated with HMW-HA, showing that cells incubated with or without HMW-HA presented equivalent DNA damage and DNA repair system activation after UVB irradiation.

UVB has multiple cellular targets that trigger different signaling cascades leading to apoptosis. Data showed that exposure of HCE cells to UVB led to the activation of caspase-3, which serves as a critical marker for apoptosis. Our data indicate that UVB-induced apoptosis was significantly decreased with HMW-HA treatment. Apoptosis is initiated via either the intrinsic pathway with caspase-9 activation or the extrinsic pathway with caspase-8. The present data report that UVB irradiation induced levels of cleaved caspase-8 and -9. UVB irradiation induced cell apoptosis via the intrinsic pathway and the extrinsic pathway. HCE cells treated with HMW-HA exhibited a significant decrease in UVB-induced capase-8 activation but few or no decrease in caspase-9 activation. The extrinsic pathway (caspase-8) is initiated through the activation of the death receptors by their respective ligands. These receptors include the tumor necrosis factor receptors, CD95 (Cluster Determinant 95)/Fas (Apoptosis-Stimulating Fragment)/APO (Apoptosis-1)-1 and the TRAIL (Tumor Necrosis Factor Related Apoptosis Inducing Ligand) receptors [24,25]. Cell apoptosis induced by such receptors occurs because of recruitment of the adaptor protein, FADD (Fas Associated Death Domain), which in turn recruits the proform of caspase-8. Aggregation of pro-caspase-8 leads to its auto-activation and subsequent activation of effector caspases such as caspase-3 [26]. HMW-HA presented neither an antioxidant effect nor DNA protective effects. It was not surprising that HMW-HA did not decrease caspase-9 activation. Indeed, phosphorylation of p53 activates the release of cytochrome c and caspase-9 via target genes such as *Bax* (BCL-2 [B-cell lymphoma-2] associated protein), *Bak* (BCL-2 homologous antagonist/killer), *Noxa* (damage), and *PUMA* (p53 upregulated modulator of apoptosis) [27]. Moreover, the GSH depletion that we observed leads to cytochrome c release and caspase-9 induction [28]. In this study, we observed a good correlation between the rise of both oxidative stress and DNA damage and caspase-9 activation. Without excluding alternative possibilities, the anti-apoptotic property of HMW-HA in our model system appears to be mediated by the inhibition of the extrinsic pathway, notably by caspase-8 inhibition.

In this study, we showed that UVB irradiation on HCE cells stimulated the release of IL-6, and IL-8. When cells were incubated with HMW-HA, production of both IL-6 and IL-8 was significantly decreased. It has been described that cytokines like IL-6 and IL-8 produced by UV-exposed cells may be associated with both inflammation and cell death [29,30]. HMW-HA decreased HCE cell death, and it could explain how HMW-HA decreased IL-6 and IL-8 observed production. However, Mitsui et al. recently observed that HA inhibits mRNA expression of proinflammatory cytokines and cyclooxygenase-2/prostaglandin E(2) production via CD44 in human fibroblasts [31]. We suggested that HMW-HA indirectly prevents IL-6 and IL-8 production in HCE cells by decreasing cell death as well as prevents cytokine release via CD44 with a direct inhibition of the inflammation process.

To our knowledge, it is the first time that the ability of HMW-HA, which is not able to absorb UVB radiation, to protect cells against UVB-induced cytotoxicity is reported. According to our results, HMW-HA provides anti-inflammatory and anti-apoptotic signals to cells exposed to UVB radiations, showing that HMW-HA is not an inert biopolymer. HCE cells express CD44 on their plasmic membranes [11]. We suggest that CD44 could be a key to understanding how HMW-HA protects HCE cells from UVB radiations.

## References

[1] Young AR (2006). Acute effects of UVR on human eyes and skin.. Prog Biophys Mol Biol.

[2] Pourzand C, Tyrrell RM (1999). Apoptosis, the role of oxidative stress and the example of solar UV radiation.. Photochem Photobiol.

[3] Genestra M (2007). Oxyl radicals, redox-sensitive signalling cascades and antioxidants.. Cell Signal.

[4] Cheng KC, Cahill DS, Kasai H, Nishimura S, Loeb LA (1992). 8-Hydroxyguanine, an abundant form of oxidative DNA damage, causes G-T and A-C substitutions.. J Biol Chem.

[5] Prives C, Hall PA (1999). The p53 pathway.. J Pathol.

[6] Rezvani HR, Mazurier F, Cario-Andre M, Pain C, Ged C, Taieb A, de Verneuil H (2006). Protective effects of catalase overexpression on UVB-induced apoptosis in normal human keratinocytes.. J Biol Chem.

[7] Okada H, Mak TW (2004). Pathways of apoptotic and non-apoptotic death in tumour cells.. Nat Rev Cancer.

[8] Weissman B, Meyer K (1954). The structure of hyalobiuronic acid and of hyaluronic acid from umbilical cord.. J Am Chem Soc.

[9] Laurent TC, Fraser JR (1992). Hyaluronan.. FASEB J.

[10] Tammi R, MacCallum D, Hascall VC, Pienimaki JP, Hyttinen M, Tammi M (1998). Hyaluronan bound to CD44 on keratinocytes is displaced by hyaluronan decasaccharides and not hexasaccharides.. J Biol Chem.

[11] Pauloin T, Dutot M, Warnet JM, Rat P (2008). In vitro modulation of preservative toxicity: High molecular weight hyaluronan decreases apoptosis and oxidative stress induced by benzalkonium chloride.. Eur J Pharm Sci.

[12] Pauloin T, Dutot M, Liang H, Chavinier E, Warnet JM, Rat P (2009). Corneal protection with high molecular weight hyaluronan against in vitro and in vivo sodium lauryl sulfate-induced toxic effects.. Cornea.

[13] Galema SA (1997). Microwave chemistry.. Chem Soc Rev.

[14] Araki-Sasaki K, Ohashi Y, Sasabe T, Hayashi K, Watanabe H, Tano Y, Handa H (1995). An SV40-immortalized human corneal epithelial cell line and its characterization.. Invest Ophthalmol Vis Sci.

[15] Borenfreund E, Puerner JA (1985). Toxicity determined in vitro by morphological alterations and neutral red absorption.. Toxicol Lett.

[16] Repetto G, del Peso A, Zurita JL (2008). Neutral red uptake assay for the estimation of cell viability/cytotoxicity.. Nat Protoc.

[17] Gomes A, Fernandes E, Lima JL (2005). Fluorescence probes used for detection of reactive species.. J Biochem Biophys Methods.

[18] Murphy G, Young AR, Wulf HC, Kulms D, Schwarz T (2001). The molecular determinants of sunburn cell formation.. Exp Dermatol.

[19] Kulms D, Zeise E, Poppelmann B, Schwarz T (2002). DNA damage, death receptor activation and reactive oxygen species contribute to ultraviolet radiation-induced apoptosis in an essential and independent way.. Oncogene.

[20] Buttke TM, Sandstrom PA (1994). Oxidative stress as a mediator of apoptosis.. Immunol Today.

[21] Chiou CC, Chang PY, Chan EC, Wu TL, Tsao KC, Wu JT (2003). Urinary 8-hydroxydeoxyguanosine and its analogs as DNA marker of oxidative stress: development of an ELISA and measurement in both bladder and prostate cancers.. Clin Chim Acta.

[22] Ziegler A, Jonason AS, Leffell DJ, Simon JA, Sharma HW, Kimmelman J, Remington L, Jacks T, Brash DE (1994). Sunburn and p53 in the onset of skin cancer.. Nature.

[23] Kraemer KH (1997). Sunlight and skin cancer: another link revealed.. Proc Natl Acad Sci USA.

[24] Haupt S, Berger M, Goldberg Z, Haupt Y (2003). Apoptosis - the p53 network.. J Cell Sci.

[25] Locksley RM, Killeen N, Lenardo MJ (2001). The TNF and TNF receptor superfamilies: integrating mammalian biology.. Cell.

[26] Thorburn A (2004). Death receptor-induced cell killing.. Cell Signal.

[27] Ishida T, Sakaguchi I (2007). Protection of human keratinocytes from UVB-induced inflammation using root extract of Lithospermum erythrorhizon.. Biol Pharm Bull.

[28] Pouzaud F, Bernard-Beaubois K, Thevenin M, Warnet JM, Hayem G, Rat P (2004). In vitro discrimination of fluoroquinolones toxicity on tendon cells: involvement of oxidative stress.. J Pharmacol Exp Ther.

[29] Caricchio R, McPhie L, Cohen PL (2003). Ultraviolet B radiation-induced cell death: critical role of ultraviolet dose in inflammation and lupus autoantigen redistribution.. J Immunol.

[30] Yoshizumi M, Nakamura T, Kato M, Ishioka T, Kozawa K, Wakamatsu K, Kimura H (2008). Release of cytokines/chemokines and cell death in UVB-irradiated human keratinocytes, HaCaT.. Cell Biol Int.

[31] Mitsui Y, Gotoh M, Nakama K, Yamada T, Higuchi F, Nagata K (2008). Hyaluronic acid inhibits mRNA expression of proinflammatory cytokines and cyclooxygenase-2/prostaglandin E(2) production via CD44 in interleukin-1-stimulated subacromial synovial fibroblasts from patients with rotator cuff disease.. J Orthop Res.

